# Periimplant Bone Resorption at the Level of Tilted Implants in SKY Fast & Fixed Restorations

**DOI:** 10.25122/jml-2020-0131

**Published:** 2020

**Authors:** Raluca Gabriela Mocanu, Laurentiu Iulian Florica, Cristina Teodora Preoteasa, Mihaela Daniela Meghea, Elena Preoteasa

**Affiliations:** 1. Department of Complete Denture, Faculty of Dental Medicine, “Carol Davila” University of Medicine and Pharmacy,Bucharest, Romania; 2. Private practice, Bucharest, Romania; 3. Department of Scientific Research Methods-Ergonomics, Faculty of Dental Medicine, “Carol Davila” University of Medicine and Pharmacy, Bucharest, Romania

**Keywords:** Dental implants, inclined implants, fixed prosthesis, all-on-four, bone apposition

## Abstract

Implant-prosthetic rehabilitation registers multiple variants, but their short- and long-term evolution has been a frequent concern. This study aimed to evaluate the peri-implant bone resorption at the level of the tilted implants in the SKY fast & fixed restorations, with reference to clinical and treatment parameters. An observational study was conducted on a convenience sample of patients with implant-prosthetic rehabilitation in one or both jaws, according to the SKY fast & fixed protocol (Bredent, Germany). Bone resorption was assessed on panoramic radiography. Other data were collected from the patient’s medical records. Thirty tilted implants were analyzed, 12 of which were in the maxilla and 18 in the mandible. After the follow-up period, both bone resorption (maximum 7 mm) and bone apposition (maximum 8 mm) were observed. There was a tendency for the resorption to be more pronounced in the mandible, in patients where tooth loss was due to periodontal disease, and when implants with length less than 16 mm were used. Resorption was statistically significantly lower when bone addition materials and membranes were used at the extraction socket, and when SKY fast & fixed rehabilitation was performed in both jaws. SKY fast & fixed implant-prosthetic technique, which involves applying a small number of implants, and a fixed prosthesis corresponding to a shortened dental arch, is a viable method of treatment that outcomes the need for complex and expensive surgical interventions, and proves to be beneficial in maintaining the optimal parameters of bone support.

## Introduction

An implant-supported fixed prosthesis is a treatment option frequently used in dental practice. Implant-based techniques that use a reduced number of implants, axial and tilted, with immediate loading, using a fixed prosthesis, initially temporary, afterward final, with shortened dental arch, passed from fear to frequent use, followed by success and satisfaction in the daily practice. Immediate loading is a frequently used method nowadays, and it does not imply any functional or esthetic issues. It is also desirable to be as accessible as possible, with an optimal quality-price ratio. Therefore, systems like All-on-Four (Nobel Biocare, Sweden) and SKY fast & fixed (Bredent, Germany) that imply a small number of implants applied in the maxillary and/or mandibular anterior areas, the distal ones being tilted, have been developed. Also, they do not necessarily require bone augmentation. These implants have the advantage that they can be loaded a few hours after the surgical intervention [1]. These treatment alternatives probably appeared as a request of the dental practitioners to counteract the conventional dentures’ lack of stability and its consequences, e.g., mastication deficiencies [2], problems that appear more frequently in the case of patients with advanced alveolar ridge resorption. They have the advantage of avoiding additional surgical interventions made for obtaining optimal bone support for the insertion of the implants in posterior areas of the jaws. Thereby, complicated procedures can be avoided, medically and financially, e.g., transposition of the inferior alveolar neurovascular bundle, internal and external sinus lift, or bone augmentation [3]. Another advantage of the method is the shortening of the total time of prosthetic treatment with obtaining an immediate restoration on the same day, which is not possible after surgeries using bone grafts in which one has to wait a minimum time of up to 6 months in order to insert implants [3, 4]. In addition to simplifying the operating protocol and shortening the total time allocated to the treatment, distal tilted implants are also meant to provide better primary stability by their more significant length and contact on an extended surface with the adjacent bone [3].

This study aimed to evaluate peri-implant bone resorption at the level of the tilted implants in the SKY fast & fixed restorations, with reference to clinical and treatment parameters.

## Material and Methods

An observational study was conducted on a convenience sample of patients treated with implant-prosthetic rehabilitation in one or both jaws, according to the SKY fast & fixed protocol (Bredent, Germany). Taking into consideration clinical factors related to extraction of the teeth and dental implant insertion, in some of the cases, bone addition was made by using Bio-Oss (Geistlich), a human bone substitute, biochemically and structurally comparable to this purpose. Its role is to stimulate local vascularization and to promote the formation of a new bone matrix in deficient areas in this regard. A resorbable membrane was used in all the cases where bone addition was used. All patients included in this study had complete documentation, i.e., clinical and imagistic aspects, before and after implant insertion. The data was collected from patients’ medical records and were represented by age, sex, etiology of teeth loss, information regarding the tilted dental implants used (length and diameter of the Bredent implants), follow-up period, and peri-implant bone resorption.

**Figure 1:**
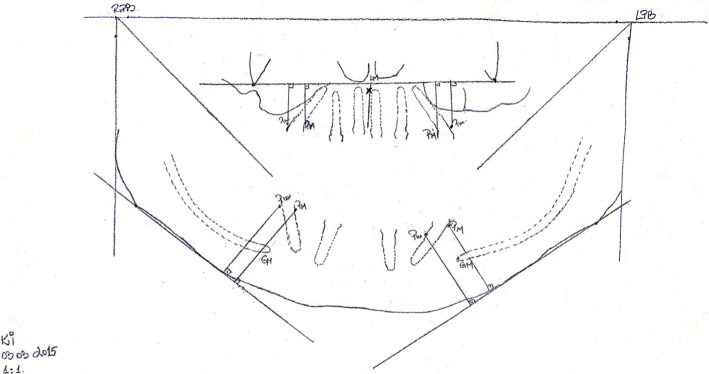
Diagram of the measurements performed.

In order to analyze peri-implant bone resorption, panoramic radiographs were used, a method that is useful and necessary in assessing long-term success [5, 6]. The assessment method was similar to that described by Puricelli [7]. The authors, following multiple research on dental models and radiographs, have developed an easy system for performing measurements on panoramic radiographs, minimizing the errors that may occur in making recordings. They proposed a graphical method of tracing the main mandibular landmarks, based strictly on panoramic radiographs, which allows the comparison of the proportions between the skeletal landmarks and the dental or implant landmarks, individually and taken as a whole. This, together with the method described by Guller et al. [8], were used for this research. In the mandible, the method implied the tracing of the following parameters: mandibular outline, horizontal reference plane, vertical reference plane, the bisector of the angle formed by the vertical and horizontal plane, the point of the bisector (right and left), the condylar point (right and left), the median line of the mandible, mental foramen, gonial point (right and left), mandible tangent, the outline of the mandibular canal (right and left). Firstly is traced the mandible outline, followed by the inferior bone borderline of the mandibular body. Afterward, the most superior and posterior point at the level of the bilateral mandibular condyle is traced, points that will generate the horizontal reference plane and the two vertical reference planes, right and left. The meeting point between the horizontal reference plane and one of the vertical reference planes (right or left) will generate two 90 degrees angles. The gonial anthropometric point and the most inferior point from the mandibular body must be traced. The mandible tangent was also traced, a plane that will serve to measure bone resorption height in the vertical plane. In the maxilla, the method implied the tracing the following: the lowest points on the orbital border (right and left), the lower outlines of the two zygomatic processes of the maxillary bones, the horizontal plane which relates de two points that mark the lower limit of the zygomatic processes, anterior nasal spine, median maxillary line, the outline of the lower edge of the two sinuses, the outline of the lower edge of the nasal fossae (Figure 1).

In the cases that were no panoramic x-rays in which the maxillary premolars were present, needed for comparison with the panoramic radiography made after implants insertion, the position of the first premolar was considered to be located next to the line tangent to the mesial limit of the infraorbital foramen and perpendicular to the plane joining the two points below the orbital borders. In the mandible, when x-rays with premolars were not available, their position was considered according to Guller et al. [8], i.e., at a distance of 35% of the horizontal dimension of the mandibular body, calculated from the level of the median line.

The evaluation of the resorption was made using panoramic x-rays performed by the same radiological center on a 1:1 scale or 1:1,25:1, a situation in which the values obtained were transformed to comply with the ratio of 1:1. Data regarding the peri-implant bone height from the premolar maxillary and mandibular areas were collected, previous to the dental implant insertion, and after the insertion and the prosthetic loading of these. When the measurement of the bone height was registered in the premolar area, the minimum period of time on the post-surgical x-rays was 4 months.

Data analysis was performed mainly by descriptive statistics, globally, and by patient subgroups. Regarding the peri-implant bone, the resorption was recorded with a positive sign and the apposition with a negative sign in the database. According to the data distribution pattern, nonparametric tests (Mann-Whitney, Kruskal-Wallis Test) were used to compare the groups. The statistical significance level used was p<0.05. 

## Results

The study was conducted on 11 patients, of which 9 were male and 2 female, aged between 54 and 85 years (median 63 years). The cause of tooth loss was related to deep periodontal damage (3 patients), due to complications of dental caries (3 patients) or mixed etiology (5 patients). Fixed prosthesis on implants using the SKY fast & fixed protocol, using tilted implants positioned distally, was applied to the maxilla (2 patients), to the mandible (5 patients) or bimaxillary (4 patients). The post-insertion follow-up period was between 4 and 76 months (median 14 months).

Correspondingly, 30 angulated integrated implant SKY fast & fixed protocols were analyzed, from which 12 were placed on the upper jaw and 18 on the lower jaw. These had a length between 10-16 mm (the majority, no=22 of 16 mm) and a diameter of 3.5 or 4mm. Bone addition was performed for the majority of the implants (n=26), and in this case, a protection membrane was also used.

**Figure 2:**
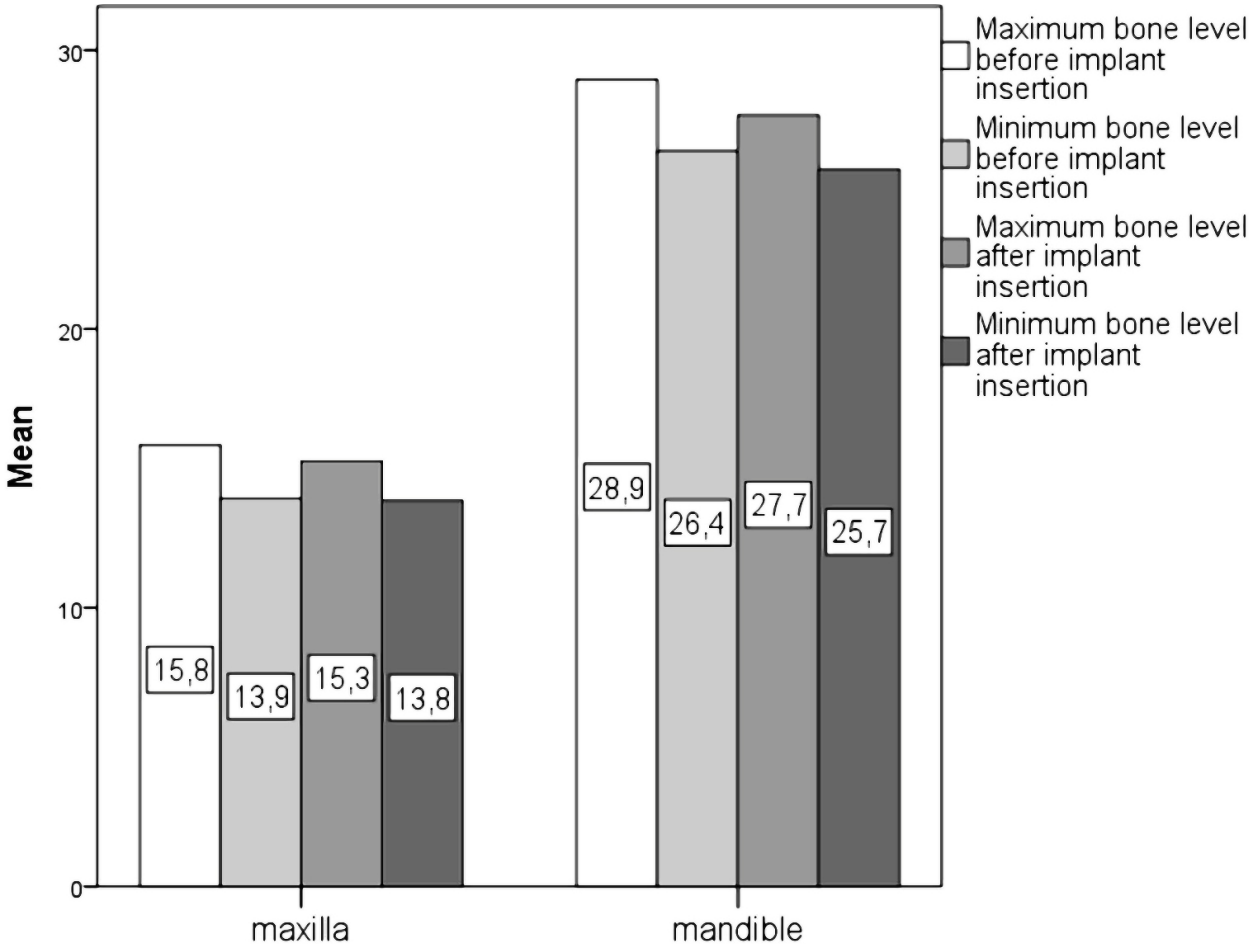
Bone level before and after tilted implant insertion.

In the case of each measurement performed, maximum and minimum values corresponding to the evaluated premolar areas or the peri-implant bone were recorded. The maximum and minimum bone height, respectively, up to the reference plane did not register significant changes before and after the implants’ insertion. A significant difference between the mandibular and maxillary bone support was noticed (Figure 2).

After follow-up, both resorption and apposition were observed. From the maximum bone level values, bone apposition ranged between 2 mm and 8 mm (mean 4.33 mm) and bone resorption ranged between 1 mm and 7 mm (mean 3.92 mm), with a mean value for all the implants of 0.7 mm. For the lowest values recorded, the bone apposition ranged between 4 mm and 8 mm (mean 6 mm), and resorption varied between 4 mm and 7 mm (mean 4.89 mm), the mean value for all implants being 0.6 mm (Table 1).

**Table 1:** Evolutionary pattern of the peri-implant bone at the level of tilted implants.

**General pattern of peri-implant bone level**	Bone level values	
	Maximum value	Mnimum value
**Implants with bone apposition **	n=6	n=2
**Implants without bone modification**	n=12	n=19
**Implants with bone resorption **	n=12	n=9

There was a tendency for the resorption to be more pronounced in the mandible when the teeth were lost due to periodontal disease, in implants with a length of less than 16 mm. However, a statistically significant difference was not recorded. Bone resorption was statistically significantly lower when bone substitutes and membranes were used, and when the SKY fast & fixed was used in both jaws (Table 2).

**Table 2:** Peri-implant bone resorption examined on tilted implants in relationship with clinical aspects and treatment particularities.

	Maximum bone resorption (mean)	p	Minimum bone resorption (mean)	p
**Tilted implants inserted in the**				
Maxilla	-0.08	0.158	0.41	0.325
Mandible	1.22		0.72	
**Tooth loss etiology**				
Periodontal disease	1.625	0.635	0.5	0.527
Caries	0		0.75	
Both causes	0.57		0.57	
**Bone addition and membrane usage**				
Yes	0.08	0.011	0.15	0.001*
No	4.75		3.5	
**SKY fast & fixed applied to the**				
Maxilla	2.25	0.011	1.5	0.001*
Mandible	3		2	
Bimaxillary	-1.135		-0.5	
**Implant length**				
16 mm	0.90	0.530	0.77	0.682
<16 mm	2		1.33	

## Discussion

All-on-4® treatment concepts and their derivatives, such as SKY fast & fixed, are accepted treatment solutions with good outcomes; researches indicated a success rate of about 95% at 10-year follow-up [9]. Among the major differences of these treatment alternatives compared to the conventional implant-supported prosthesis is the insertion of distal implants in an inclined position to bypass anatomical landmarks, such as the mandibular canal and the inferior alveolar nerve in the lower jaw, or the maxillary sinuses in the upper jaw [10, 11]. This essential feature of differentiating All-on-four and SKY fast & fixed from conventional treatment plans has been the subject of extensive debate and research over the years to demonstrate and balance the benefits and risks of the tilting used on the distal implants. Most studies have focused on the correlation between the size of the distal extension of the permanent prosthetic restorations and the degree of angulation and how they act on the bone dimensions. Thus, forces similar to those developed at the occlusal level were applied during functional movements in different areas of the final restorations; therefore, the way how the pressure influenced the peri-implant bone was analyzed. It was concluded that as the angulation increases, the length of the distal extension decreases, which will lead to lower tensions in the cortical bone, in the implants and the prosthetic restoration. Most of these studies were conducted by experimental methods, mainly by the finite element analysis that allowed the simulation of models with 4 or 5 implants of which the distal ones were tilted at different angles, from 0 degrees to 45 degrees [12-14].

In this research, the vast majority of implants were inserted with bone substitutes’ aid into the extraction site. In most cases, these methods were applied because of the severe periodontal disease or complications of caries, frequently presenting periapical lesions. In the case of all implants included in the present study, the Bredent surgical protocol that implies inserting the distal implants at an angulation from 30 to 45 degrees, and 16 mm length was used. There were cases in which bone structure could not support this kind of length, and a shorter one was needed. The insertion of the distal implants inclined at an angle between 30-45 degrees allows the use of distal extensions of up to two teeth without negatively modifying the peri-implant bone support. The bone augmentation techniques had favorable results in most patients, with cases of bone apposition and resorption as well, and with a vast majority of bone growth in contrast with those that did not receive a bone augmentation. Both in the case of implants with bone augmentation and the case of those without bone augmentation, the minimum and maximum values of the peri-implant bone were recorded at different periods, values measured from the alveolar ridge to the tangent of the mandible and to the horizontal plane joining the two points that mark the lower limit of the zygomatic processes of the maxilla. Thus, the following values were obtained: the maximum value of the maxillary bone height was 19 mm, while in the mandible, significantly higher values of up to 42 mm were recorded. There were also significant differences in the minimum bone values recorded for the two arches. If in the lower jaw the minimum value of the bone height was not below 15 mm in any implant, in the upper jaw, there were situations where the bone height did not exceed the value of 12 mm, a significant difference between the two values, the reason for which the bone augmentation procedures also were mainly performed in the maxilla. The average values of bone height were 14.95 mm in the maxilla and 26.69 mm in the mandible, which strengthened the idea that the mandibular bone is an easier option than the maxillary bone when it comes to the surgical procedure.

All the values stated above were also compared with measurements performed prior to surgical therapy in the premolar area, an area favorable for the insertion of tilted implants. As in the previous case, the highest bone height value - 36 mm - was recorded in the mandible, a value significantly higher than in the upper arch where the maximum value of the recordings was 20 mm. When it comes to the average values of the maxillary and mandibular hard bone support, the following measurements were obtained: 14.84 mm and 27.6 mm. Compared to the initial situation, prior to dental extractions, it can be said that the average value of bone resorption, a value obtained by comparing the initial vertical bone dimensions and values at different periods after the implant insertion and the prosthetic loading, was 4.29 mm with a maximum value of 7 mm at 4 years after the initial surgical procedures. Compared to the success criteria for conventionally placed implants, 9 implants registered higher bone resorption than the value considered optimal in relation to the period [15]. However, this reference may not be suitable for tilted implants in this concept, given the design and positioning differences when considering the long-term impact on the treatment’s success. At the same time, there are studies that provide evidence that marginal bone loss in tilted and axial implants is no different [16, 17]. 

Given the fact that most patients involved in the study also needed to increase the bone supply through addition techniques, the values obtained were also compared with the situation in which patients did not need to use bone substitutes. Thus, in the case of those with bone addition, 23.07% of the implants registered bone apposition at the level of the alveolar ridge, with a maximum value of 8 mm, and an average value of 4.2 mm. Bone resorption of various degrees was registered in only 30.76% of the analyzed cases, with a maximum value of 7 mm and an average value of 3.33 mm. According to the results of this study, there is lower peri-implant bone resorption when this treatment concept is used bimaxillary. A possible explanation would be that in unimaxillary restorations, there are higher occlusal forces, especially when the antagonists are natural teeth.

The main limitation of the present study is the small number of cases included. However, it can be used as a starting point for future broader studies on the same topic or topics derived from the context presented above. Also, measurements made on panoramic radiographs can associate limitations in evaluating bone support, being recommended to confirm these results through research that uses three-dimensional imaging as the evaluation method.

## Conclusion

Given the limitations of the research, the results suggest that bone resorption in tilted implants in the SKY fast & fixed treatment concept is relatively low. At the same time, it seems that there is a more favorable evolution when bone addition with the bone substitute is used in the extraction socket, and when the treatment solution is applied bimaxillary. The SKY fast & fixed implant-prosthetic technique, which involves applying a small number of implants, and a fixed prosthesis corresponding to a shortened dental arch, is a viable method of treatment that outcomes the need for complex and expensive surgical interventions, and proves to be beneficial in maintaining the optimal parameters of the bone.

## Conflict of Interest

The authors declare that there is no conflict of interest.

## Acknowledgments

All authors had an equal contribution as the first author.

## References

[1] Maló P, Rangert B, Dvärsäter L (2000). Immediate function of Brånemark implants in the esthetic zone: a retrospective clinical study with 6 months to 4 years of follow-up. Clin Implant Dent Relat Res.

[2] Oncescu Moraru AM, Preoteasa CT, Preoteasa E. (2019). Masticatory function parameters in patients with removable dental prosthesis. J Med Life.

[3] Malo P., de Araújo Nobre M, Lopes A, Moss SM, Molina GJ (2011). A longitudinal study of the survival of All-on-4 implants in the mandible with up to 10 years of follow-up. J Am Dent Assoc.

[4] Preoteasa E., Florica LI, Obadan F, Imre M, Preoteasa CT, Turkyilmaz I (2015). Minimally Invasive Implant Treatment Alternatives for the Edentulous Patient-Fast & Fixed and Implant Overdentures. Current Concepts in Dental Implantology.

[5] Xie Q., Soikkonen K, Wolf J, Mattila K, Gong M, Ainamo A (1996). Effect of head positioning in panoramic radiography on vertical measurements: an in vitro study. Dentomaxillofac Radiol.

[6] Humphries S., Devlin H, Worthington H (1989). A radiographic investigation into bone resorption of mandibular alveolar bone in elderly edentulous adults. J Dent.

[7] Puricelli E. (2009). Panorametry: suggestion of a method for mandibular measurements on panoramic radiographs. Head Face Med.

[8] Güler AU, Sumer M, Sumer P, Biçer I (2005). The evaluation of vertical heights of maxillary and mandibular bones and the location of anatomic landmarks in panoramic radiographs of edentulous patients for implant dentistry. J Oral Rehabil.

[9] Soto-Penaloza D., Zaragozí-Alonso R, Penarrocha-Diago M, Penarrocha-Diago M (2017). The all-on-four treatment concept: Systematic review. J Clin Exp Dent.

[10] Adell R., Eriksson B, Lekholm U, Brånemark PI, Jemt T. (1990). Long-term follow-up study of osseointegrated implants in the treatment of totally edentulous jaws. Int J Oral Maxillofac Implants.

[11] De Rossi M, Santos CM, Migliorança R, Regalo SC (2014). All on Four® fixed implant support rehabilitation: a masticatory function study. Clin Implant Dent Relat Res.

[12] Friberg B., Sennerby L, Linden B, Gröndahl K, Lekholm U (1999). Stability measurements of one-stage Brånemark implants during healing in mandibles. A clinical resonance frequency analysis study. Int J Oral Maxillofac Surg.

[13] Bevilacqua M., Tealdo T, Menini M (2011). The influence of cantilever length and implant inclination on stress distribution in maxillary implant-supported fixed dentures. J Prosthet Dent.

[14] Ekelund JA., Lindquist LW, Carlsson GE, Jemt T (2003). Implant treatment in the edentulous mandible: a prospective study on Brånemark system implants over more than 20 years. Int J Prosthodont.

[15] Albrektsson T., Zarb G, Worthington P, Eriksson AR (1986). The long-term efficacy of currently used dental implants: a review and proposed criteria of success. Int J Oral Maxillofac Implants.

[16] Del Fabbro M, Ceresoli V (2014). The fate of marginal bone around axial vs. tilted implants: a systematic review. Eur J Oral Implantol.

[17] Monje A., Chan HL, Suarez F, Galindo-Moreno P, Wang HL (2012). Marginal bone loss around tilted implants in comparison to straight implants: a meta-analysis. Int J Oral Maxillofac Implants.

